# Case Report: Optic Atrophy and Nephropathy With m.13513G>A/MT-ND5 mtDNA Pathogenic Variant

**DOI:** 10.3389/fgene.2022.887696

**Published:** 2022-06-03

**Authors:** Valentina Barone, Chiara La Morgia, Leonardo Caporali, Claudio Fiorini, Michele Carbonelli, Laura Ludovica Gramegna, Fiorina Bartiromo, Caterina Tonon, Luca Morandi, Rocco Liguori, Aurelia Petrini, Rachele Brugnano, Rachele Del Sordo, Carla Covarelli, Manrico Morroni, Raffaele Lodi, Valerio Carelli

**Affiliations:** ^1^ Department of Biomedical and Neuromotor Sciences (DIBINEM), Alma Mater Studiorum-University of Bologna, Bologna, Italy; ^2^ IRCCS Istituto delle Scienze Neurologiche di Bologna, Bologna, Italy; ^3^ Nephrology Division, “S. Giovanni Battista Nuovo” Hospital, Foligno, Italy; ^4^ Department of Nephrology and Dialysis, S. Maria della Misericordia Hospital, Perugia, Italy; ^5^ Department of Medicine and Surgery, Section of Anatomic Pathology and Hystology, Medical School, University of Perugia, Perugia, Italy; ^6^ Department of Experimental and Clinical Medicine, Section of Neuroscience and Cell Biology, School of Medicine, Università Politecnica delle Marche, Ancona, Italy

**Keywords:** LHON, Mitochondrial nephropathy, m.13513G>A mutation, MT-ND5, cerebellum

## Abstract

Isolated complex I deficiency represents the most common mitochondrial respiratory chain defect involved in mitochondrial disorders. Among these, the mitochondrial DNA (mtDNA) m.13513G>A pathogenic variant in the NADH dehydrogenase 5 subunit gene (MT-ND5) has been associated with heterogenous manifestations, including phenotypic overlaps of mitochondrial encephalomyopathy with lactic acidosis and stroke-like episodes, Leigh syndrome, and Leber’s hereditary optic neuropathy (LHON). Interestingly, this specific mutation has been recently described in patients with adult-onset nephropathy. We, here, report the unique combination of LHON, nephropathy, sensorineural deafness, and subcortical and cerebellar atrophy in association with the m.13513G>A variant.

## Introduction

Mitochondrial diseases (MDs) constitute a heterogenous group of rare diseases characterized by frequent multisystemic involvement and pleomorphic clinical manifestations. Mitochondria are crucial for tissues and organs with a high energy demand, including the central nervous system and in particular the neural retina and organs such as the kidney and liver (La Morgia et al., 2020). Primary kidney disorders in MDs are more frequently reported in children than in adults, and their typical manifestations include both tubular disorders and glomerulopathies (Emma et al., 2016).

The most important causes of focal segmental glomerulosclerosis (FSGS) are represented by defects in coenzyme Q10 biosynthesis due to nuclear DNA mutations and the m.3243A>G mutation affecting the mtDNA-encoded tRNA-Leu gene (Emma et al., 2016). Kidney involvement was recently described in association with SSBP1 gene mutations, leading to mtDNA depletion and expressing a complex phenotype, which may include optic atrophy, foveopathy and retinal dystrophy, sensorineural deafness, myopathy, and kidney failure, requiring transplantation (Del Dotto et al., 2020).

Mitochondrial nephropathy has also been described in patients with the m.13513G>A pathogenic variant in the MT-ND5 gene encoding for a complex I subunit (Bakis et al., 2020).

Complex I deficiency is the most common biochemical defect in MDs (Alston et al., 2017), and pathogenic mutations have been identified in all seven mtDNA-encoded subunits. Among these, the MT-ND5 gene is a mutational “hot-spot” (Liolitsa et al., 2003; Bannwarth et al., 2013), and its pathogenic variants have been associated with different clinical phenotypes, including Leigh syndrome (LS) (Kirby et al., 2003; Blok et al., 2007), mitochondrial encephalomyopathy with lactic acidosis stroke-like episode (MELAS) syndrome (Liolitsa et al., 2003; Shanske et al., 2008), Leber’s hereditary optic neuropathy (LHON) (Howell et al., 1993), or combinations of these (Wang et al., 2010).

Here, we report a case with the unique combination of optic atrophy, sensorineural deafness, subcortical and cerebellar atrophy, and adult-onset nephropathy associated with the heteroplasmic m.13513G>A/MT-ND5 mtDNA variant.

## Case Description

**FIGURE 1:**
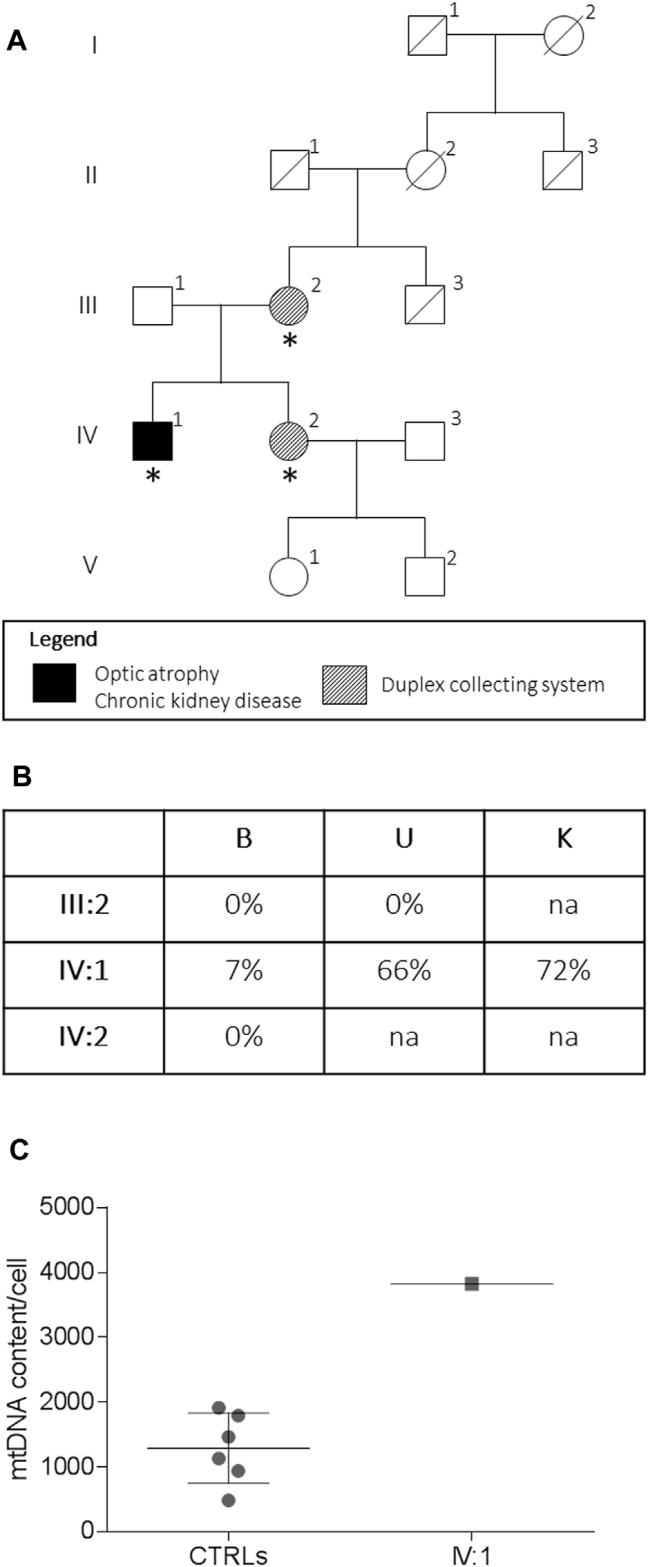
Pedigree and genetic analysis. Pedigree of the family **(A)**. Asterisks indicate the individual investigated. The m.13513G > A/MT-ND5 variant heteroplasmy **(B)** in the peripheral blood **(B)**, urinary sediment (U), and kidney biopsy (K) is reported. The mtDNA content from the kidney **(C)** from the proband (IV:1, *n* = 1) and controls (CTRLs, *n* = 6). Data were presented as a scatter plot with mean and SD.

A 48-year-old male patient was referred to our institution with a history of bilateral visual loss and chronic kidney disease (CKD). His mother and sister were affected by an asymptomatic duplex collecting system (Figure 1). From age 43, he presented bilateral sensorineural deafness. At the age of 47, he developed acute-onset visual loss in the right eye, followed by the left eye after a few months. Ophthalmological examination showed visual acuity loss (0.3 in OD and 0.5 in OS). Due to the LHON-like phenotype, the mtDNA was analyzed for classic primary mutations, which were negative. MtDNA complete sequencing from muscle biopsy revealed the m.13513G>A/MT-ND5 heteroplasmic mutation. Therefore, idebenone treatment (900 mg/die) was started, about 10 months after the onset of visual loss in the first eye. The patient reached the nadir 15 months after the onset (visual acuity was counting fingers bilaterally). At the last follow-up 26 months after the onset, the patient showed partial recovery of visual acuity (0.1 in OD and 0.06 in OS), and therapy with idebenone is still ongoing.

**FIGURE 2:**
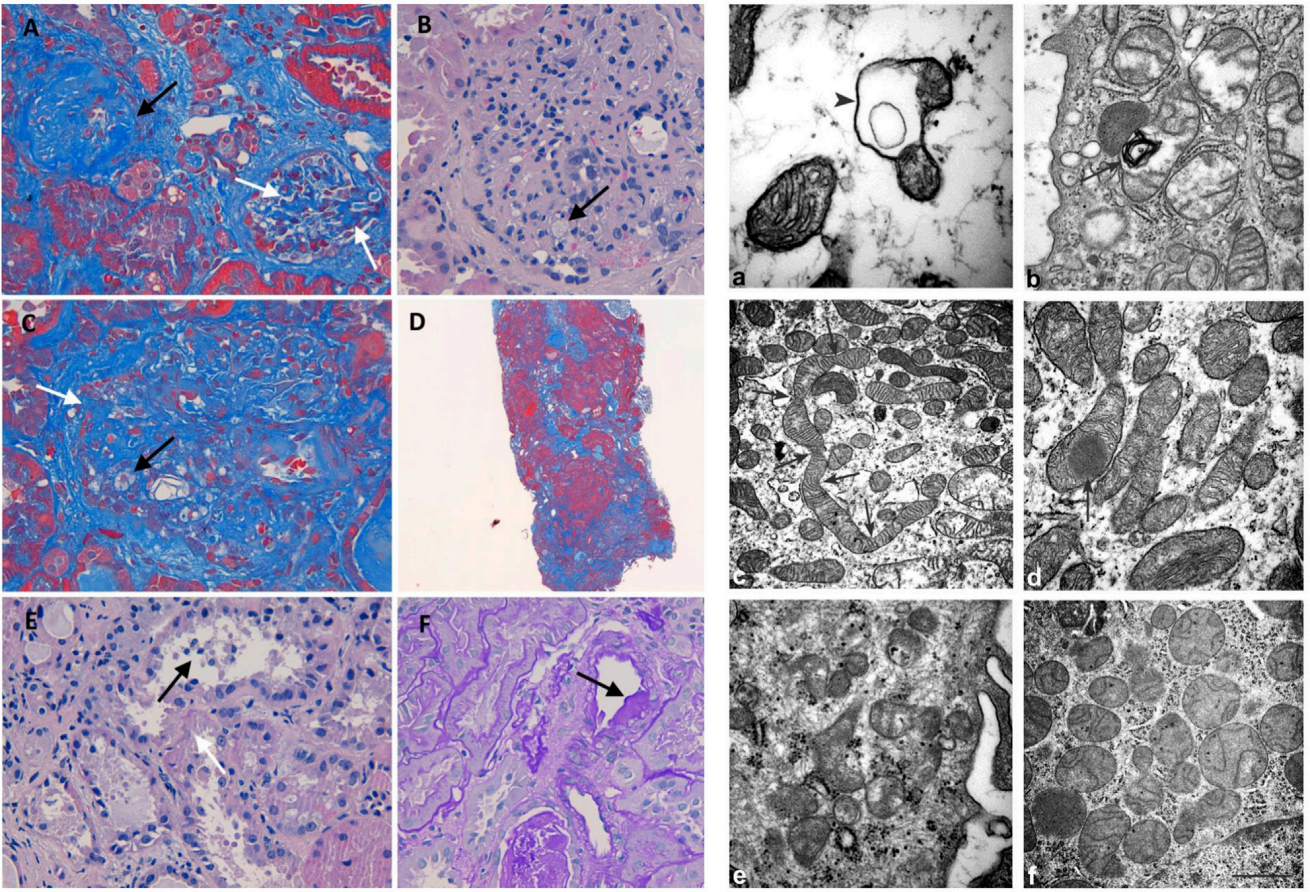
Histology and electron microscopy. Masson’s trichrome (200x): global (black arrow) and segmental (white arrows) glomerulosclerosis **(A)**. H&E (400x): glomerulus with mesangial matrix expansion, mesangial hypercellularity, capillary loops adherent to Bowman capsule, and foam cells (black arrow) **(B)**. Masson’s trichrome (400x): foam cells (black arrow) and segmental (white arrows) sclerosis **(C)**. Masson’s trichrome (400x): diffuse interstitial fibrosis **(D)**. H&E (400x): loss of apical cytoplasm, necrosis of individual cells, detachment of epithelial cells in tubular lumen (black arrow), denudation of the tubular basement membrane (white arrow) **(E)**. PAS (400X): hyalinosis in a small artery (black arrow) **(F)**. Mitochondrion with a cyst-like structure (arrowhead) in **(a)** and a myelin figure (arrow) in **(b)**. In **(c)**, the arrows represent the narrowing of a long mitochondrion and in **(D)** an electron-dense inclusion within a mitochondrion. The kidney of control: normal mitochondria contained in glomerulus **(e)** and proximal tubule **(f)** Scale bar: a = 0,5 µm; b = 0.45 µm; c = 1.25 µm; d = 1.25 µm; e = 0,5 µm; f = 1 µm

The patient also presented a five-year history of secondary hypertension and CKD, for which he was taking a low-salt, low-protein diet. He never developed tubulopathy signs. Renal biopsy specimens showed FSGS (Figure 2). Autoimmune and cardiovascular diseases and Fabry and Alport syndromes were excluded. The patient is on dialysis since September 2021 and is currently on a waiting list for a kidney transplant.

**FIGURE 3:**
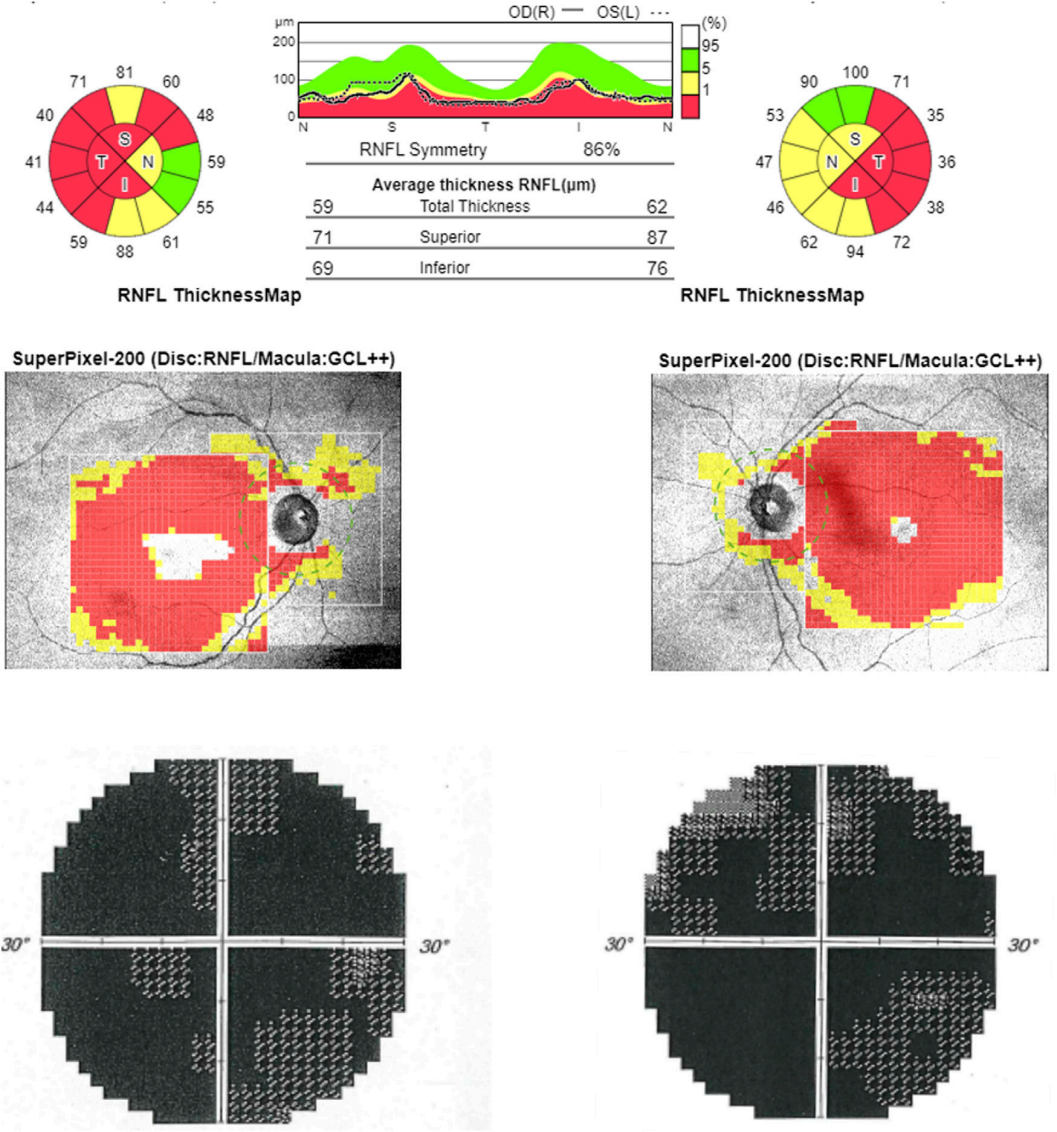
Optical coherence tomography (OCT). OCT (upper line) shows bilateral RNFL thinning more evident in the temporal sector. Computerized visual fields (lower line) show a generalized defect bilaterally.

At the time of our first observation, neurologic examination showed a marked reduction of visual acuity bilaterally, upper limb dysmetria, and diffuse brisk deep tendon reflexes. The Mini-Mental State Examination (MMSE) score was 28 (a possible influence of low vision should be considered). Fundus examination revealed the temporal optic disc pallor to be more severe in the right eye. Optical coherence tomography (OCT) disclosed a bilateral diffuse and severe thinning of the retinal nerve fiber layer (Figure 3). Blood examinations showed normal lactic acid levels in plasma at rest (9.6 mg/dl, normal range 5–22) but abnormally elevated levels after standardized exercise (55.1 mg/dl) and after recovery (36.7 mg/dl). The serum creatinine value was elevated (4.61 mg/dl, normal range 0.5–1.2). Electrocardiogram was unremarkable.

## Materials and Methods

### Renal Biopsy Histology and Electron Microscopy

Renal biopsy was performed for light microscopy and electron microscopy without samples for immunofluorescence. Hematoxylin and eosin (H&E), periodic acid Schiff (PAS), Masson’s trichrome, Jones Methenamine Silver stains, and CD68 immunohistochemistry (clone Kp-1, ready to use, Cell Marque) were applied for light microscopy.

### Genetic Analysis

DNA was extracted from peripheral blood and urine sediment by using the Maxwell^®^ 16 Blood DNA Purification Kit. DNA from formalin-fixed paraffin-embedded tissues (FFPE) was purified starting from three sections of 10 microns each, using the NucleoSpin Tissue kit (Macherey-Nagel, Dueren, Germany). The m.13513G>A/MT-ND5 variant was quantified by the SNaPshot Multiplex System (Álvarez-Iglesias et al., 2008). MtDNA content/cell was assessed by Real-Time PCR (Giordano et al., 2014).

### Brain MRI Protocol

The high-field (3T) protocol included 3D T1- 3D FLAIR T2 axial TSE T2 and SWI imaging and a single-voxel proton MR spectroscopy (1H-MRS) study of the left parietal white matter.

## Results

### Histology and Electron Microscopy

Light microscopy examination revealed eight glomeruli. Three glomeruli were globally sclerotic. Masson’s trichrome staining disclosed three glomeruli with segmental glomerulosclerosis (Figure 2A). Another glomerulus, beyond sclerosis, showed mesangial matrix expansion, mesangial hypercellularity, and capillary loops adherent to Bowman capsule and foam cells (Figures 2B,C). The reactivity of foam cells for CD68 was consistent with that of macrophages. Diffuse tubular atrophy and interstitial fibrosis and mononuclear inflammation involved 40% of the cortex (Figure 2D). Some tubular segments showed a thyroidization pattern and focal acute proximal tubular injury (Figure 2E). Hyalinosis was present in a small artery (Figure 2F). These histological features were consistent with FSGS (Columbia classification) (Man-Hoon Han, 2019). At electron microscopy, we observed in the podocytes some mitochondria containing cyst-like structures (Figure 2a), while in proximal tubules, there were a variety of mitochondrial alterations (Figures 2b−d), including myelin figures apparently lying in the mitochondrial matrix, long narrowed mitochondria, electron-dense inclusions, mitochondria with scarce cristae arranged on the periphery (not shown), and giant mitochondria (not shown).

### Genetic Analysis

The assessment of the m.13513G>A/MT-ND5 variant in the peripheral blood, the urinary sediment, and the kidney biopsy from the proband revealed a variable load of mutant heteroplasmy (7, 66, and 72%, respectively) (Figure 1C), whereas the mutation was absent in the tissues from the asymptomatic mother and sister. The mtDNA content from the kidney was increased compared to that of controls (Figure 1C).

### Brain MRI

**FIGURE 4:**
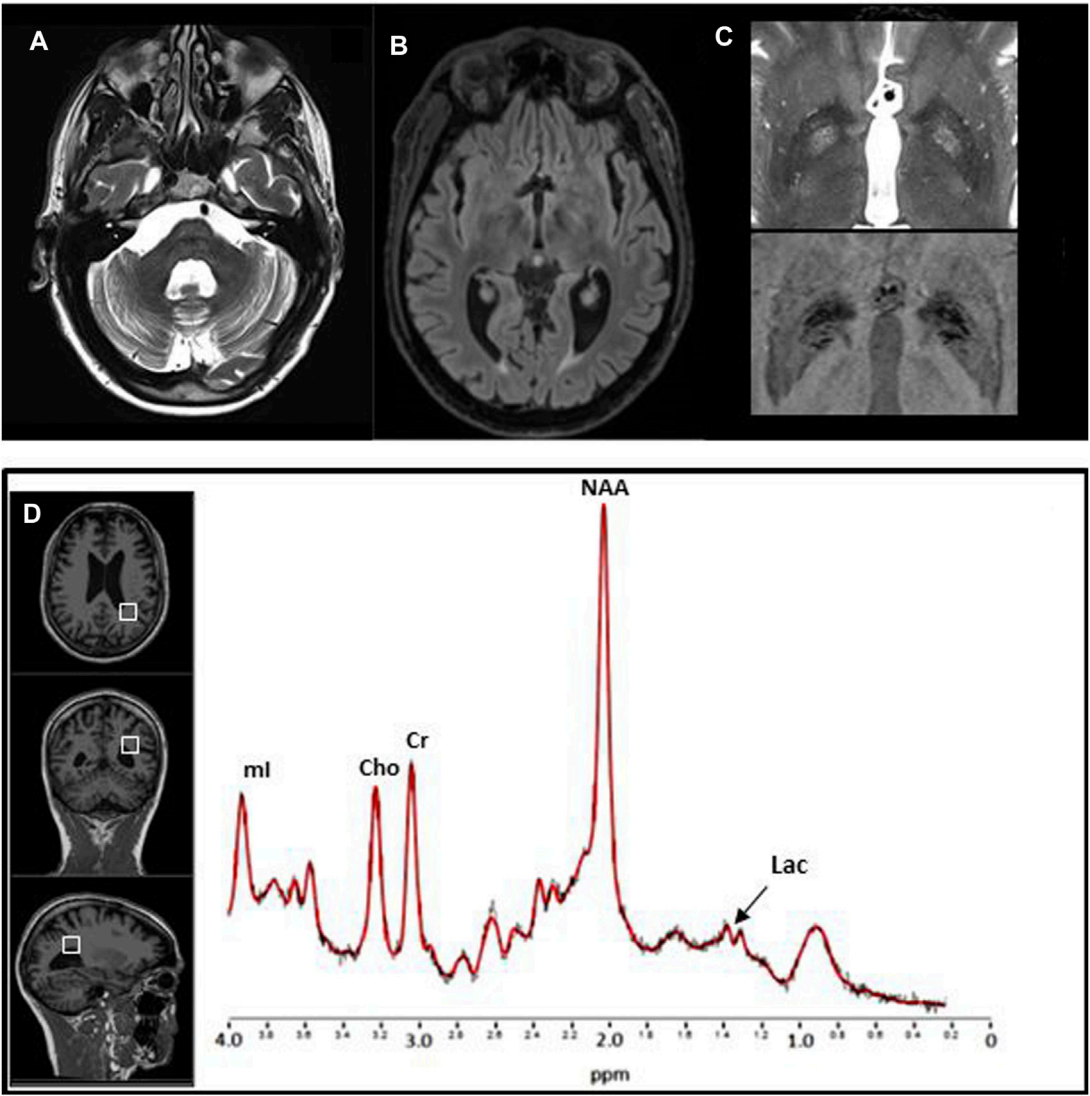
Brain MRI and proton MR spectroscopy study. Brain MRI. Axial TSE T2 **(A)** and FLAIR T2 **(B)** weighted images show, respectively, IV ventricle dilation and lateral ventricle occipital horn dilation associated with periventricular hyperintensity. On axial TSE T2, bilateral symmetrical hyperintense signal changes in the globi pallidi [**(C)** upper image], with correspondent hypointensity on the axial SWI [**(C)** lower image], consistent with mineralization, is evident. **(D)** 1H MR spectrum (3T Siemens Skyra scanner, TR/TE/NS = 2000 ms/30 ms/64) from the volume of interest (8 ml) in the left parietal white matter (on the left the localization on 3D T1 images) shows the resonance at 1.3 ppm coming from pathological traces of lactate (Lac). NAA = N-acetyl aspartate, Cho = choline, Cr = creatinine, mI = myo-inositol.

Brain MRI detected mild optic nerve atrophy, diffuse subcortical cerebral atrophy, enlargement of IV ventricle, and moderate enlargement of pericerebellar subarachnoid spaces. Pallidal nuclei signal changes consistent with mineralization were present, as confirmed by a CT scan. Single-voxel proton MR spectroscopy study of the parietal white matter revealed, compared to sex and age-matched healthy controls, normal N-acetyl aspartate (NAA), choline (Cho), and myo-inositol (mI) content, but traces of lactate, which normally is absent (Figure 4).

## Discussion

We, here, reported the unprecedented combination of LHON-like optic neuropathy, sensorineural deafness, subcortical and cerebellar atrophy, and nephropathy as a new phenotypic manifestation of the m.13513G>A/MT-ND5 pathogenic variant. The kidney pathology, characterized by histologic evidence of glomerular and tubular cell involvement and abnormal mitochondrial ultrastructure, was associated with high heteroplasmic mutation load.

The m.13513G>A/MT-ND5 variant, first described by Santorelli et al. in a patient with MELAS (Santorelli et al., 1997), has been later identified in over 40 additional individuals with heterogenous clinical manifestations, including LS (Shanske, et al., 2008), Wolff Parkinson-White (WPW) syndrome (Wang et al., 2008), and LHON and overlapping phenotypes (Santorelli et al., 1997). We expanded this spectrum by presenting a case which, besides LHON, had nephropathy, sensorineural deafness, and subcortical and cerebellar atrophy, features commonly seen in MELAS (Gramegna et al., 2021).

This mutation leads to the substitution of an evolutionarily conserved amino acid (D393N) in the ND5 subunit of complex I, the entry site of the mitochondrial respiratory chain, with consequent impairment of oxidative phosphorylation. Usually, the heteroplasmic mutant load shows a good correlation with disease severity and the spectrum of clinical phenotypes, ranging from fatal neonatal forms to asymptomatic cases (Ng et al., 2018).

In addition to optic atrophy, other ophthalmologic findings have been described in association with the m.13513G>A/MT-ND5 variant, such as ptosis, ophthalmoplegia, early-onset cataract, and even central retinal vein occlusion (Chol et al., 2003; Kirby et al., 2003; Sudo et al., 2004; Ruiter et al., 2007; Hsieh et al., 2011). However, only one LHON case without other extraocular manifestations has been reported so far (Sun et al., 2021).

Remarkably, this pathogenic variant has also been recently described in patients with adult-onset nephropathy (Motoda et al., 2012; Ng et al., 2019; Bakis et al., 2020). Another reported case associated with nephropathy and optic atrophy has been reported (Bakis et al., 2020), with a different nucleotide change, yet affecting the same codon, but leading to a different amino acid substitution (D393G *vs*. D393N) (Corona et al., 2001). The kidney histology and heteroplasmy were not available.

Our case not only presented with classic LHON but also suffered bilateral sensorineural deafness and asymptomatic CKD preceding the neurological manifestation. The kidney biopsy showed nonspecific histological features (granular swollen tubular cells, arterioles, and small arteries with disorganized arrangement and irregular size of smooth muscle cells), consistent with hyalinosis, which is usually reported in hypertensive patients. However, electron microscopy revealed abnormal mitochondrial morphology both in podocytes and proximal renal tubules. The mtDNA analysis confirmed a high m.13513G>A/MT-ND5 mutant load in the kidney supporting that histological and ultrastructural abnormalities were consequent to mitochondrial dysfunction (Finsterer and Scorza, 2017). Furthermore, the heteroplasmic load of mutant mtDNA was remarkably low in blood cells, as previously reported for other cases with MT-ND5 mutations (Valentino et al., 2006; Sun et al., 2021), and the genetic analysis in proband’s asymptomatic mother and sister failed to reveal the pathogenic mtDNA change, which may suggest that the duplex collecting system could be an independent trait.

According to the previously reported cases, kidney involvement with the m.13513G>A/MT-ND5 variant can either manifest as tubulointerstitial kidney disease, sometimes with “secondary” FSGS, or segmental glomerulosclerosis. However, in all cases, glomerular mitochondria were reported to be morphologically normal (Ng et al., 2019; Bakis et al., 2020). Tubular disorders are more common in mitochondrial cytopathies, probably due to the very high metabolic rates and abundant mitochondria in renal tubular cells (Emma and Salviati, 2017). In our patient, the histological prevalence of glomerulopathy might be due to the high heteroplasmic load. The numerous mitochondria in glomerular podocytes and their crucial role in cellular functions support the glomerulosclerosis occurring in our case.

Multisystem involvement is a common hallmark of mitochondrial disease. In fact, in addition to the kidney, retinal ganglion cells, a preferential target of complex I alterations including MT-ND5 mutations, also were affected in this patient, leading to the LHON-like phenotype (Carelli et al., 2009), suggesting, as for the kidney, a supra-threshold load of mutant heteroplasmic mtDNA.

In conclusion, we reported the clinical, radiologic, histologic, and genetic data of this case with the unique clinical combination of nephropathy, deafness, subcortical and cerebellar atrophy, and LHON due to the m.13513G>A/MT-ND5 pathogenic variant. This case expands the range of clinical manifestations and highlights the new phenotypic overlap of both glomerular and tubular involvement in the kidney. Our case and the others raise the possibility that the kidney is a preferential tissue target of the m.13513G>A/MT-ND5 pathogenic variant. We are also aware that this mutation may be a more frequent cause of LHON than previously recognized, and the association of FSGS and optic atrophy must prompt testing of the m.13513G>A/MT-ND5 variant.

## Data Availability

The datasets for this article are not publicly available due to concerns regarding participant/patient anonymity. Requests to access the datasets should be directed to the corresponding author.
